# Cellular consequences of small supernumerary marker chromosome derived from chromosome 12: mosaicism in daughter and father

**DOI:** 10.1590/1414-431X2022e12072

**Published:** 2022-06-22

**Authors:** M.O. Freitas, A.O. dos Santos, L.S. Barbosa, A.F. de Figueiredo, S.P. Pellegrini, N.C.K. Santos, I.S. Paiva, A. Rangel-Pozzo, L. Sisdelli, S. Mai, M.G.P. Land, M.G. Ribeiro, M.C.M. Ribeiro

**Affiliations:** 1 Laboratório de Genética, Instituto de Puericultura e Pediatria Martagão Gesteira, Universidade Federal do Rio de Janeiro, Rio de Janeiro, RJ, Brasil; 2 Programa de Pós-Graduação de Clínica Médica, Faculdade de Medicina, Universidade Federal do Rio de Janeiro, Rio de Janeiro, RJ, Brasil; 3 Serviço de Genética Médica, Instituto de Puericultura e Pediatria Martagão Gesteira, Universidade Federal do Rio de Janeiro, Rio de Janeiro, RJ, Brasil; 4 Faculdade de Medicina, Universidade do Grande Rio, Rio de Janeiro, RJ, Brasil; 5 Faculdade Medicina, UNIFESO (Centro Educacional Serra dos Orgãos), Rio de Janeiro, RJ, Brasil; 6 CancerCare Manitoba Research Institute, University of Manitoba, Cancer Care Manitoba, Winnipeg, Manitoba, Canada; 7 Laboratório de Bases Genéticas de Tumores de Tireóide, Divisão de Genética, Departamento de Morfologia e Genética, Universidade Federal de São Paulo, São Paulo, SP, Brasil; 8 Programa de Pós-Graduação em Saúde Materno Infantil, Instituto de Puericultura e Pediatria Martagão Gesteira, Universidade Federal do Rio de Janeiro, Rio de Janeiro, RJ, Brasil; 9 Laboratório NUMPEX-BIO, Campus Duque de Caxias, Universidade Federal do Rio de Janeiro, Rio de Janeiro, RJ, Brasil

**Keywords:** Small supernumerary marker chromosomes, Chromosomal instability, Molecular cytogenetics, Genotype-phenotype correlation, Telomeres

## Abstract

Constitutional genomic imbalances are known to cause malformations, disabilities, neurodevelopmental delay, and dysmorphia and can lead to dysfunctions in the cell cycle. In extremely rare genetic conditions such as small supernumerary marker chromosomes (sSMC), it is important to understand the cellular consequences of this extra marker, as well the factors that contribute to their maintenance or elimination through successive cell cycles and phenotypic impact. The study of chromosomal mosaicism provides a natural model to characterize the effect of aneuploidy on genome stability and compare cells with the same genetic background and environment exposure, but differing in the presence of sSMC. Here, we report the functional characterization of different cell lines from two familial patients with mosaic sSMC derived from chromosome 12. We performed studies of proliferation dynamics, stability, and variability of these cells using fluorescent *in situ* hybridization (FISH), sister chromatid exchanges (SCE), and conventional staining. We also quantified the telomere-related genomic instability of sSMC cells using 3D telomeric profile analysis by quantitative-FISH. sSMC cells exhibited differences in the cell cycle dynamics compared to normal cells. First, the sSMC cells exhibited lower proliferation index and higher frequency of SCE than normal cells, associated with a higher level of chromosomal instability. Second, sSMC cells exhibited more telomeric-related genomic instability. Lastly, the differences of sSMC cells distribution among tissues could explain different phenotypic repercussions observed in patients. These results will help in our understanding of the sSMC stability, maintenance during cell cycle, and the cell cycle variables involved in the different phenotypic manifestations.

## Introduction

Constitutional genomic imbalances have an important role in many human diseases. They are known to cause malformations, disabilities, neurodevelopmental delay, and dysmorphia (1). It is assumed that the phenotypic manifestation is associated with the genomic segment involved, the parental origin, and the genetic background (2).

Some clinical conditions are associated with specific genomic imbalances. Individuals with the same imbalance present similar phenotypes, while other imbalances are uncommon and the phenotypic association has not yet been recognized (3,4).

Genomic imbalances may influence morphogenesis resulting in phenotypic anomalies. During development, these imbalances are subjected to strong selective pressure to correct the anomaly, resulting in the mosaicism appearance with a normal cell lineage (5,6). General mosaicism is defined by the presence of two or more cell lines with different genotypes in the entire organism. To be considered as general mosaicism, the mosaicism should be present before the differentiation starts (7). It is assumed that small supernumerary marker chromosomes (sSMC) may appear as the result of partial trisomy rescue process in a trisomic zygote (5,8).

At a cellular level, genomic imbalances such as aneuploidy and microsatellite instability (MSI), and changes in chromosome structure (duplication, deletion, and translocation) are markers of genomic or chromosomal instability (CIN) (9).

Genomic imbalances are associated with dysfunctions in cell division and delay in the cell cycle, which can result in cell death. Also, they are associated with alterations in morphogenesis and neoplastic processes (6,10,11).

Individuals with abnormalities involving chromosome 12, such as complete trisomy of 12 and Pallister Killian syndrome (extra isochromosome 12p), are viable only with mosaicism, and the abnormal cells appear only in tissues other than blood (12,13). Detection in live births, when the mosaicism does not prevent the physiological function of normal cells, have been rarely reported (12,13). The regions considered critical to phenotype are 12q24.21-q24.23 (14); 12p13.2-pter; and 12p13.1-p13.33 (15).

All the processes that normal cells go through to become CIN precursors remains unclear, but replication stress and mitotic errors are major sources of structural and numerical CIN, respectively (16). If, on the one hand, replication stress can lead to mitotic defects and consequently aneuploidy, on the other hand, the structural CIN and aneuploidy, created by mitotic defects, may generate replication stress and DNA damage as a vicious circle (20). The evaluation of sister chromatid exchanges (SCE) is considered a very sensitive method for the detection of spontaneous chromosomal instability (17).

In extremely rare genetic conditions such as sSMC(18), it is important to understand the cellular consequences of this extra marker, as well the factors that contribute to their maintenance or elimination through successive cell cycles and phenotypic impact. The presence of sSMC and the characterization of sSMC architecture can influence: i) cell proliferation speed, which can be different in each cell line (19); ii) loss of the sSMC; iii) frequency of each cell line in different tissues; and iv) the stability of sSMC, which depends on the presence of essential structural elements, including a functional centromere and two telomeres (20). Furthermore, information about stability, viability, and variability distribution of sSMC contributes to a better understanding and faster diagnosis of this type of chromosomal anomaly.

The occurrence of chromosomal mosaicism provides a natural model to characterize the effect of aneuploidy on genome stability. The comparison of cells with the same genetic background, which differ only by the genomic region involved in the imbalance, contributes to the characterization of the alteration of cellular processes and responses to the presence of anomalous chromosomal material during cell division (21,22). This characterization, together with the evaluation of tissue imbalance distribution, could clarify their role in different phenotypic manifestations (15).

Here, we report the functional characterization in two relatives (father and daughter), both with sSMC derived from chromosome 12, which is found in mosaic constitution, with a normal cell line and the distribution of these cells in several tissues. The functional evaluation presented here includes the comparison of proliferation dynamics, cell stability in culture, and sister chromatid exchange between normal and sSMC cells. In addition, we quantified the telomere-related genomic instability of these cells using 3D telomeric profile analysis by quantitative fluorescence *in situ* hybridization (Q-FISH).

## Material and Methods

### Patients

Two patients with a chromosomal mosaicism (mos47,X_,+mar/46,X_) and with the sSMC derived from chromosome 12 were evaluated. Patient 1 belongs to a cohort of 19 patients with uncharacterized marker chromosomes evaluated at Cytogenetic Laboratory of Instituto de Puericultura e Pediatria Martagão Gesteira (IPPMG), UFRJ, Brazil. Patient 1 presented several clinical abnormalities. Patient 2 (patient 1's father) has no phenotypic alterations. The informed consent was obtained from both patients (approved by the Ethics Committee of IPPMG/UFRJ No. 13/09).

### Constitutional analysis

Metaphase cells from cultured peripheral blood were evaluated by G-banding and FISH with whole chromosome painting (wcp), α-satellite, and LSI probes. FISH was performed using commercial probes: D12Z1(12p11.1-q11.1) (Cytocell aquarius, Cytocell, UK); wcp 12 (Chromoprobe multiprobe octachrome, Cytocell, *ETV6* (12p13.2); TelG (telomere, repeats of TTAGGG) (DAKO, Denmark) according the manufacturers' instructions.

To analyze distribution of sSMC in different tissues, we performed FISH using commercial D12Z1(12p11.1-q11.1) probes in uncultivated interphase cells of peripheral blood, oral mucosa, and urinary sediment samples without culture. The samples were washed with saline solution and centrifuged (1000 *g*, room temperature, 5 min). The pellet was resuspended with Carnoy’s fixative solution (one part of glacial acetic acid and three parts of methanol). Subsequently, the suspension obtained was dripped onto silanized slides for the FISH technique. The frequencies were compared between the two different cell lines from the same patient (intrapatient analysis) and among the equal cell lines from different patients (patients 1 and 2) (interpatient analysis) using Pearson’s chi-squared test.

The aCGH was performed in peripheral blood by a commercial laboratory using a high-resolution genomic screening platform (Agilent^®^ Platform - CGH+SNP array 400K, pipeline_CGH_v.5.1.2, Brazil). The exam detects copy number variation (CNV), segments larger than 100Kb, and selected single nucleotide polymorphism (SNP). The analyzed data were based on the reference genome GRCh37.

### Functional analysis

#### Frequency of sSMC cells

To evaluate the *in vitro* selection and proliferation speed of cell lines with sSMC, the frequency of cells with sSMC was evaluated in blood cultures of 48, 72, and 96 h, counting the amount of sSMC cells in 100 cells. For the intrapatient statistical analysis, the frequencies were compared using Pearson’s chi-squared test. For the interpatient analysis, the frequencies were compared using Fisher’s test.

#### BrdU incorporation

In the beginning of the culture, 10 µg/mL BrdU (Sigma, USA) was added and the culture was incubated for 72 h. The culture was processed following standard protocols. Differential staining was obtained by the technique modified from previous studies (23,24).

#### Replication dynamic analysis

For the replication dynamics of cell lines with sSMC, the proliferation index of each cell line was established counting the frequency of cells in first, second, and third *in vitro* divisions after incorporating BrdU into a 72-h culture. Fifty metaphases from each cell line were visualized and classified as first, second, or third division (cells at first division have 100% of chromatids stained, cells at second division have 50% of chromatids stained, and cells in third division have around 25% of chromatids stained). The proliferation index was (No. of cells at first division x1) + (No. of cells at second division x2) + (No. of cells at third division x3) / total number of cells analyzed (25). The proliferation indices were compared using the Levene test for the analysis of variance followed by the *t*-test.

#### Sister chromatid exchange analysis

To evaluate chromosomal stability, the frequency of SCE between normal and sSMC cell lines was compared. Twenty second division metaphases from each cell line were analyzed and the amount of SCE was counted for each cell. Cell instability was measured by the average of SCE in each cell line (amount of SCE / No. of metaphases analyzed) and the results between normal cell line and sSMC cell line were compared (26). For the intrapatient statistical analysis, the averages of SCE were compared using Kolmogorov-Smirnov and Shapiro-Wilk normality tests followed by Mann-Whitney U test. For the interpatient statistical analysis, the averages of SCE were compared using the *t*-test, after Kolmogorov-Smirnov and Shapiro-Wilk normality tests and the Levene test for the analysis of variance.

#### Q-FISH analysis

Q-FISH was performed to evaluate 3D telomere organization between normal and sSMC cell lines according to the previous protocol published by Knecht et al. (27). Commercial telomere (DAKO) and D12Z1 (Cytocell Aquarius) probes were used in the analysis. Fifty cells from each cell line were imaged using a Zeiss AxioImager Z2 microscope equipped with a Zeiss AxioCamMRmm Rev 3 camera (Carl Zeiss Canada Ltd., Canada). The Cy3 filter was used at a constant exposure time (335 ms), while exposure time for the DAPI filter varied. The images were captured in 60 z-stacks at 200-nm intervals to create 3D images of the cell nuclei. The images were then deconvolved using Zen Blue software (Carl Zeiss Canada Ltd., Canada), exported to .tiff files and analyzed using TeloView^®^ v1.03 program (Telo Genomics Corp., Canada) (28,29)]. Six different parameters were measured by TeloView^®^: number of telomere signals; total intensity of signals and average intensity of signals (proportional to telomere length); number of aggregates (cluster of telomeres found in close proximity to each other that, at 200 nm optical resolution, cannot be further resolved as separate entities); *a/c* ratio (cell cycle distribution into G0/G1, S, or G2 phases, according to the position of the telomeres in the cell nuclei; the higher the *a/c* ratio, the greater the proportion of cells in proliferations); and nuclear volume (computed by summing the volumes of each individual image in the x, z, and y dimensions - volumes were measured taking into account slice thickness). The parameters were compared between the two different cell lines from the same patient and among the equal cell lines from different patients (patients 1 and 2) using a nested factorial analysis of variance followed by a least-square means multiple comparison.

**Figure 1:**
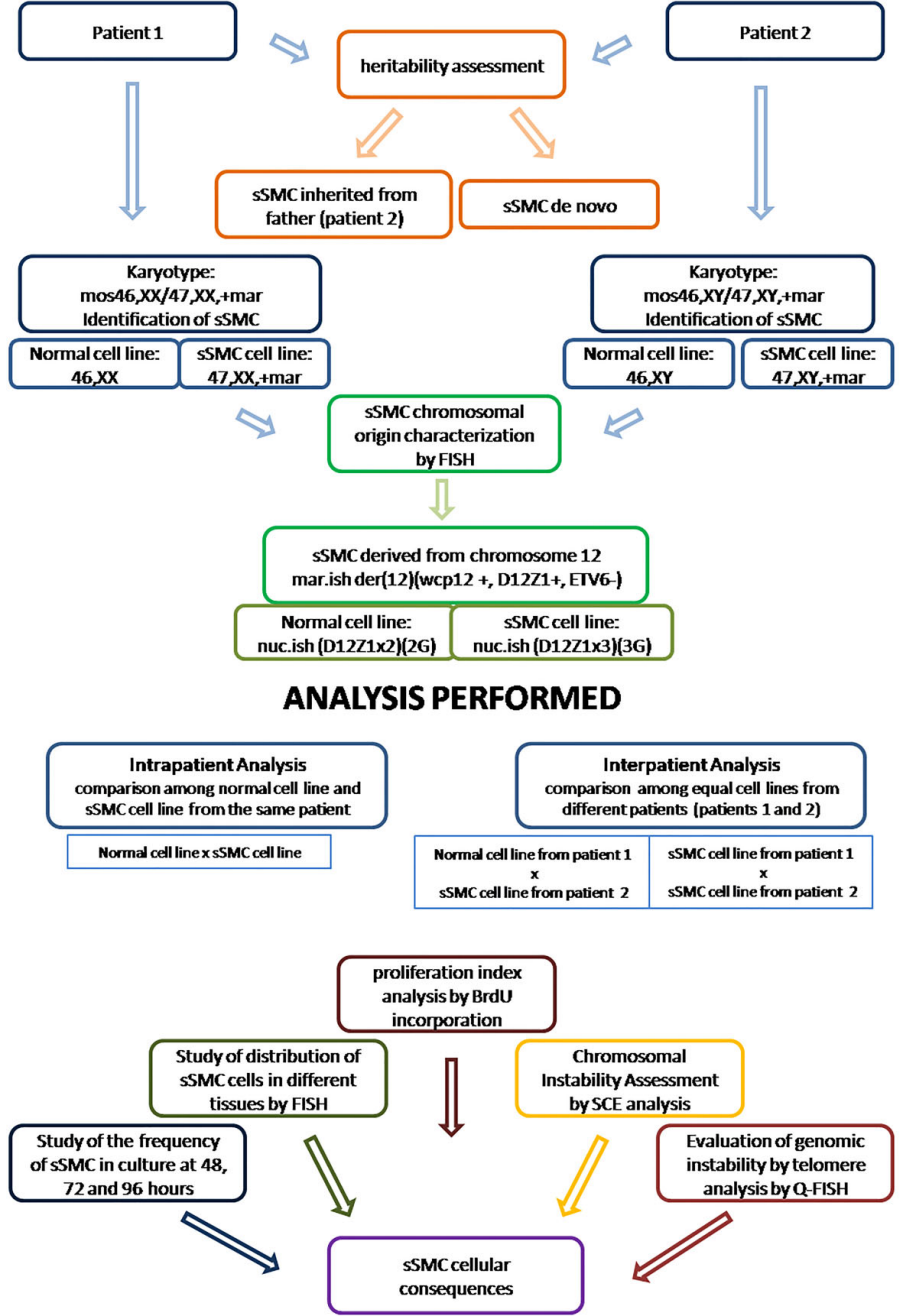
Flowchart showing the performed analysis. Metaphase cells from cultured peripheral blood from patients 1 and 2 were evaluated by G-banding and a mosaic small supernumerary marker chromosome (sSMC) was identified. FISH was performed for sSMC chromosomal origin characterization. Normal cell line karyotype was 46,X_,nuc.ish(D12Z1x2) and sSMC cell line karyotype was 47,X_,+mar.ish der(12)(wcp12+,D12Z1+,ETV6-).nuc.ish(D12Z12x3). Frequency and distribution analysis of sSMC cells, proliferation index, sister chromatid exchange, and 3D telomere organization analyses were performed to evaluate the impact of the presence of sSMC.

A flowchart showing all the performed analyses is shown in Figure 1.

## Results

### Description of patients

*Patient 1*: A 12-year-old girl, daughter of non-consanguineous parents (mother was 33 and father was 38 years old), was referred to the Genetics Service of IPPMG at the age of five years due to language delay, auditory conduction changes, epilepsy, skin pallor, attention and concentration difficulties, and autistic spectrum features. Global developmental delay was confirmed. There was a maternal family history of delayed speech and learning difficulties. On physical examination at the age of seven years, she presented skin pallor, macrostomia, wide forehead and high hair line, a downturned nasal tip, protruding ears, fetal pads on fingers 2, 3, 4, and 5 of the right hand and 4 and 5 of the left hand, syndactyly of toes 2 and 3, and joint hypermobility.

*Patient 2*: Patient 2 was a 38-year-old man, patient 1's father, and son of non-consanguineous parents. There was no family history of delayed speech, learning difficulties, or genetic syndromes. On physical examination, patient 2 did not show any phenotypic remark.

### Constitutional analysis

#### sSMC chromosomal origin

**Figure 2:**
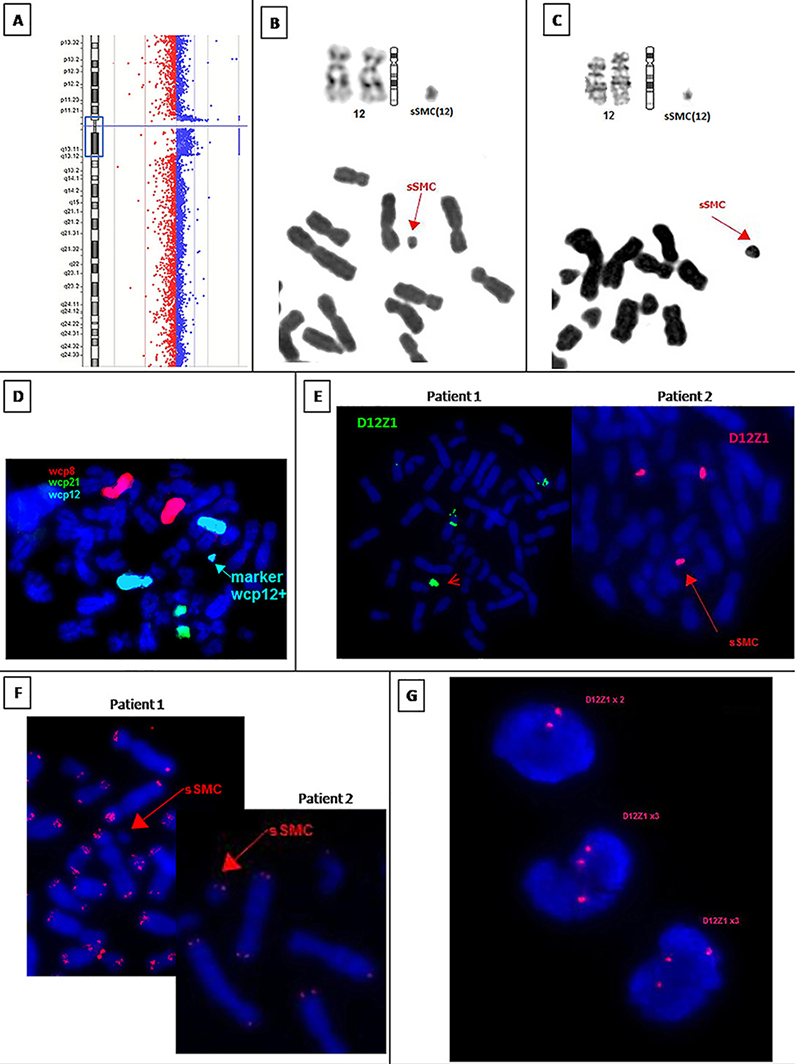
A, Patient 1's array comparative genomic hybridization results showing gain of bands 12p11.21 and 12q13.11. **B**, Patient 1's partial karyotype in G-banding, showing both normal chromosome 12 and small supernumerary marker chromosomes (sSMC), and partial metaphase in conventional staining showing the linear shape of sSMC. **C**, Patient 2's partial karyotype in G-banding, showing normal chromosome 12 and sSMC, and partial metaphase in conventional staining showing the linear shape of sSMC. **D**, Patient 1's partial metaphase from FISH with chromosome painting showing sSMC and both normal chromosome 12 in Aqua, 2 normal chromosomes 21 in green, and 2 normal chromosomes 8 in red. **E**, FISH metaphase with D12Z1 probe in patients 1 and 2, showing the sSMC indicated by a red arrow. **F**, FISH with DNA probe specific to telomeric repeats showing absence of telomeric repeats cluster in patient 1's sSMC and cluster of telomeric repeats in patient 2's sSMC. **G**, FISH uncultivated peripheral blood cells with two or three copies of D12Z1 probe. Normal cell line means two copies of D12Z1 probe while sSMC cell line means three copies of D12Z1 probe.

*Patient 1*: Initial chromosome analysis showed karyotype mos47,XX,+mar(6)/46,XX(24). Conventional staining showed a linear sSMC that did not participate in satellite association (Figure 2B), present in 32% of analyzed cells (one hundred cells). FISH analysis showed that the marker chromosome was a sSMC derived from chromosome 12, causing partial chromosome trisomy as can be seen on Figure 2D and E. The mother presented a normal karyotype and the father (patient 2) presented the same sSMC in 18% of the blood cells analyzed (one hundred cells). After FISH, the karyotype was redefined to mos47,XX,+mar.ish der (12)(wcp12+,D12Z1+,ETV6-,TelG-)pat/46,XX. No cluster of telomeric repeats was revealed in sSMC with FISH of labeled (TTAGGG)n repeat (TelG) (Figure 2F). The array comparative genomic hybridization (aCGH) (performed commercially) (Figure 2A), showed results -arr[GRCh37] 12p11.21q13.11(32598017_48639744)x2∼3 - confirming the partial trisomy of the proximal region of chromosome 12 between the bands 12p11.21 and 12q13.11.

*Patient 2*: Initial cytogenetic analysis showed a karyotype: mos47,XY,+mar(3)/46,XY (27) (Figure 2C). Conventional staining showed a linear sSMC with same size and morphology of his daughter’s sSMC (patient 1), present in 18% of cells analyzed (one hundred cells) (Figure 2C). The sSMC origin was confirmed by FISH analysis with wcp12 probe and D12Z1 (Figure 2E). After FISH, the karyotype was redefined to mos47,XY,+mar.ish der (12)(wcp12+,D12Z1+,ETV6-,TelG+)/46,XY. FISH with DNA probe specific to telomeric repeats (TelG) showed cluster of telomeric repeats in sSMC (Figure 2F). The aCGH to investigate duplication of 12p11.21q13.11 and sSMC (performed commercially) showed no copy number variation. Cytogenetic analysis of patient 2's parents showed normal karyotype in both.

#### Distribution of sSMC cells in different tissues

**Figure 3:**
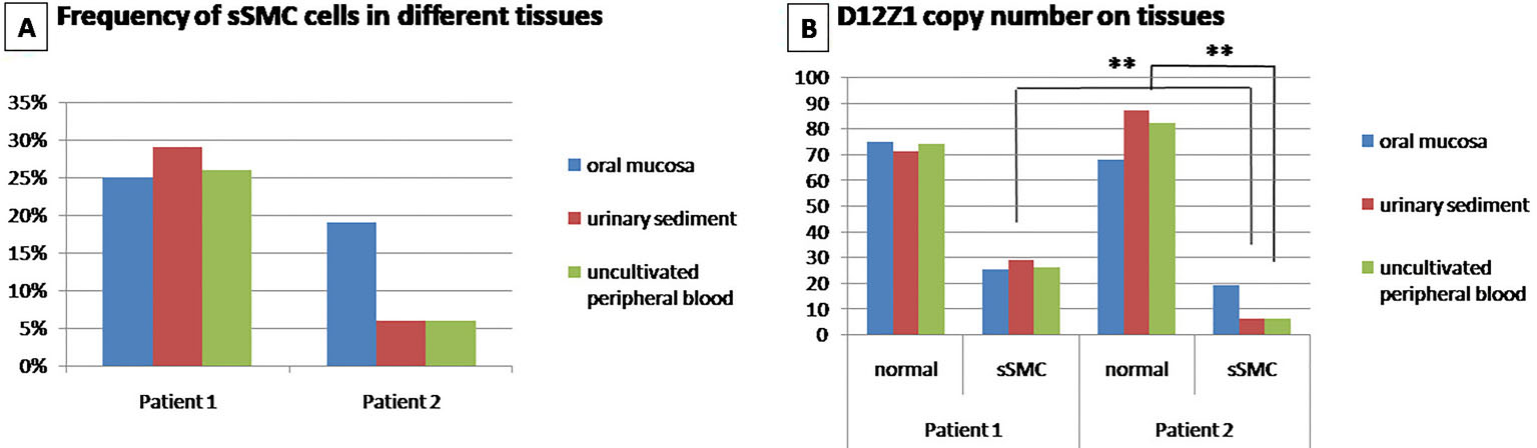
A, Frequencies of cells with small supernumerary marker chromosomes (sSMC) in uncultivated different tissues. **B**, Number of cells with two or three copies of D12Z1 in each tissue (normal cell line means two copies of D12Z1 while sSMC cell line means three copies of CEP12 probe). **P<0.05, Pearson chi-squared test.

The frequency of interphase cells with sSMC for each analyzed tissue (oral mucosa, urinary sediment, and uncultivated peripheral blood) is described in Table 1 and shown in Figure 3A. The number of cells with two (normal cells) or three copies (sSMC cells) of D12Z1 probe from each uncultivated tissue is shown in Figure 3B.

**Table 1:** Frequency of interphase small supernumerary marker chromosomes (sSMC) cells in each uncultivated tissue of patient 1 and 2 and statistical analysis.

Patient	Oral mucosa	Urinary sediment	Uncultivated blood	P value
1	25%	29%	26%	0.801
2	19%	6%	6%	**0.004**
P value (Patient 1 *vs* 2)	**0.001**	**<0.001**	**<0.001**	

Bold type indicates statistically significant differences (Pearson chi-squared test).

There was no significant difference in frequency of sSMC cells between the analyzed tissues on patient 1. In patient 2, the frequency of sSMC cells in uncultivated blood was significantly higher compared with the other two analyzed tissues (oral mucosa and urinary sediment) (P=0.004).

In addition, patient 1 presented a significantly higher frequency of cells with sSMC in all tissues compared with patient 2 (P<0.001). Photographs of normal cells and sSMC cells in FISH with D12Z1 are shown in Figure 2G.

### Functional analysis

#### Frequency of sSMC

**Figure 4:**
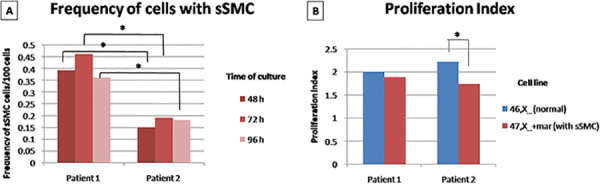
A, Frequency of small supernumerary marker chromosomes (sSMC) cells in cultures with different durations (48, 72, 96 h). **B**, Proliferation index of different cell lines from both patients (normal cells and sSMC cells). *P<0.05, Pearson chi-squared test and *t*-test.

The frequency of cells with sSMC in patient 1 was 39% at 48 h, 46% at 72 h, and 36% at 96 h; for patient 2, it was 15% at 48 h, 19% at 72 h, and 18% at 96 h. The number of cells with sSMC was significantly higher in patient 1 compared to patient 2 at all culture times. In addition, patient 2 had significantly more normal cells than patient 1. The comparison of frequencies of sSMC cells at different times between patients is shown in Figure 4A.

#### Dynamics of proliferation analysis

For both patients (patient 1 and 2), the proliferation index was higher in the normal cell line (2 and 2.22, respectively) compared with sSMC cell line (1.88 and 1.74, respectively), but this difference was statistically significant only for patient 2 (P=0.005), as shown in Figure 4B. The proliferation index from each cell line is described in Table 2.

**Table 2:** Proliferation index (PI) and frequency of mean sister chromatid exchanges (SCE) from both cell lines (normal and with small supernumerary marker chromosomes (sSMC)) of patients 1 and 2 and statistical analysis.

Patient	Cell line	Proliferation index	P value (PI) (*t*-test)	Frequency of SCE	P value (SCE) (Mann-Whitney U test)
1	Normal	2	0.442	1.7	**<0.001**
	sSMC	1.88		4	
2	Normal	2.22	**0.005**	2.6	0.075
	sSMC	1.74		3.45	

Bold type indicates statistically significant differences.

#### Sister chromatid exchange

**Figure 5:**
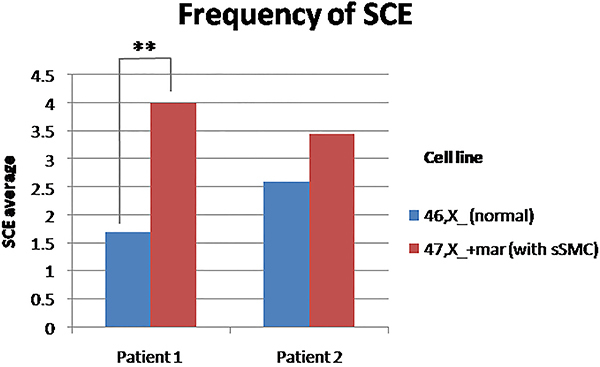
Average frequency of sister chromatid exchanges (SCE) from the two cell lines [normal and with small supernumerary marker chromosomes (sSMC)] of patients 1 and 2. **P<0.05, Mann-Whitney U test.

**Figure 6:**
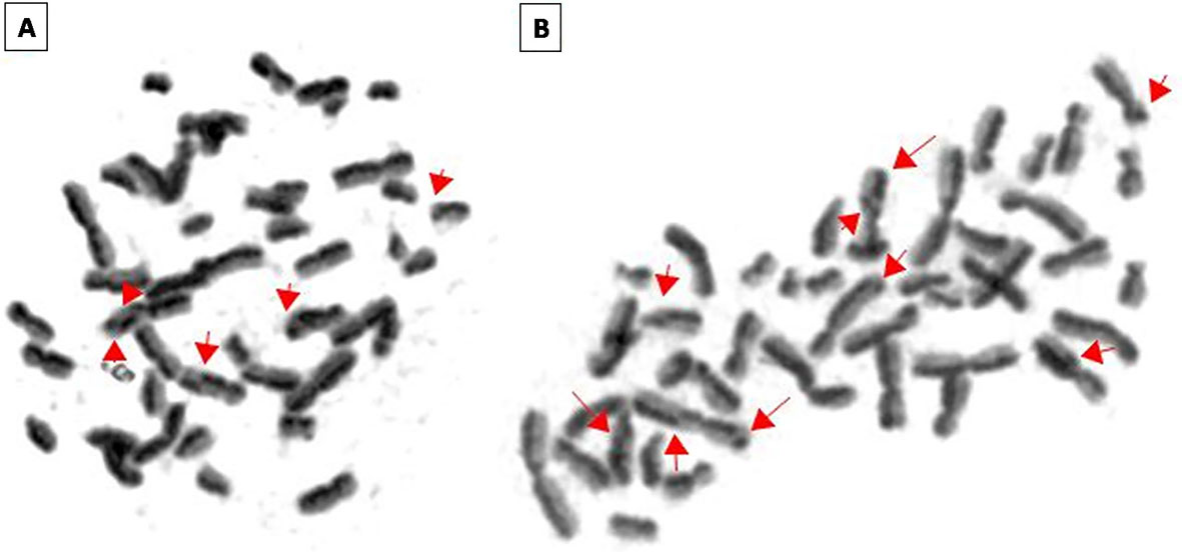
Normal cell line metaphases of patients 1 (**A**) and 2 (**B**) in second division with sister chromatid exchanges indicated by red arrows.

For both patients (1 and 2), the frequency of SCE was higher on sSMC cell line (4 and 3.45, respectively) than normal cell line (1.7 and 2.6), but this difference was statistically significant only for patient 1 (P<0.001)(Figure 5). Photographs showing SCE are shown in Figure 6 and the average of SCE frequency is shown in Table 2.

#### Genomic instability - telomere analysis

**Figure 7:**
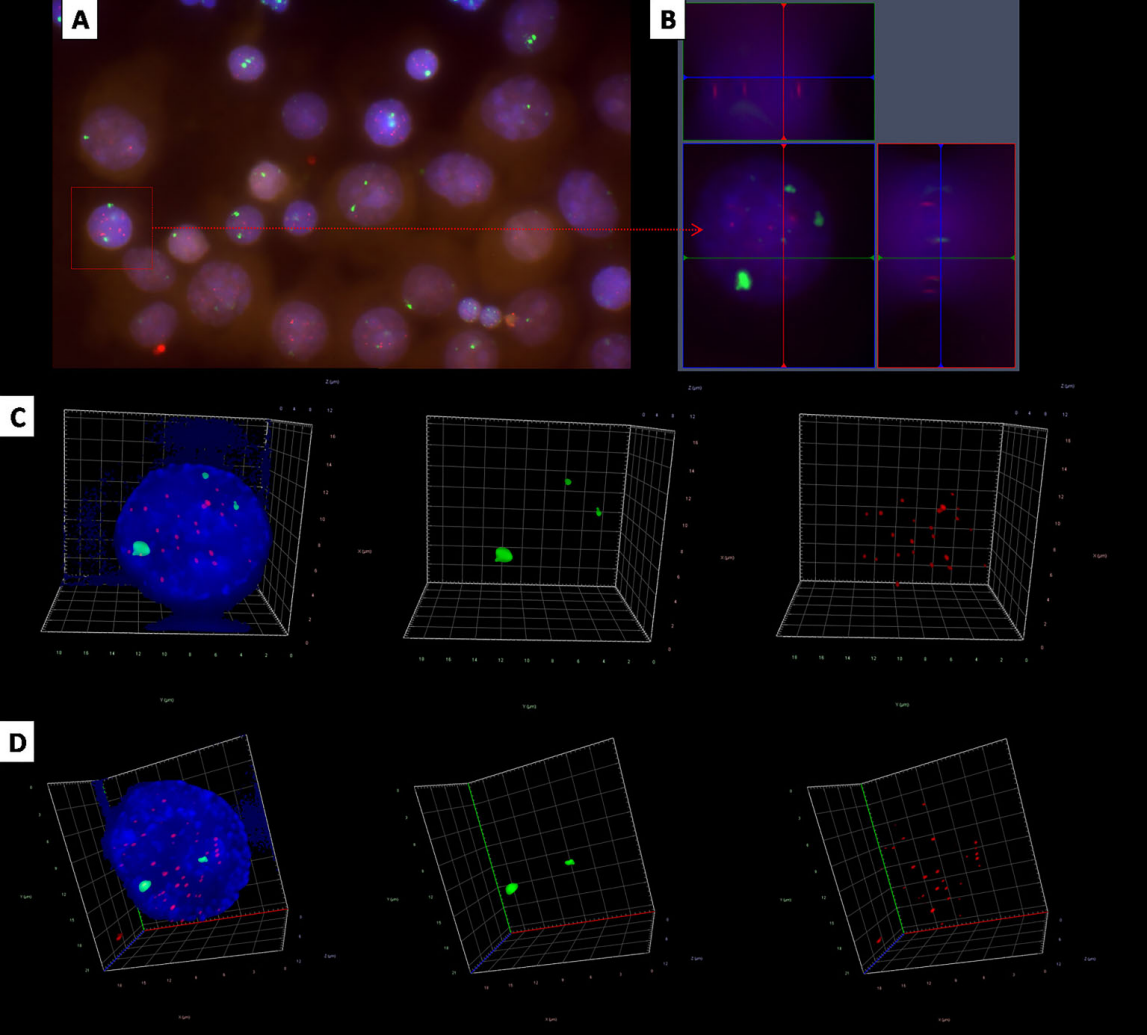
Representative images from quantitative fluorescence *in situ* hybridization (Q-FISH). **A**, 2D raw image; **B**, 2D image separated by X, Y, and Zdimensions; **C**, 3D deconvolved nuclei image of a small supernumerary marker chromosomes (sSMC) cell and representative 3D nuclear telomere distribution (red signals) with and without the counterstained nucleus (blue); **D**, 3D deconvolved nuclei image of a normal cell and representative 3D nuclear telomere distribution (red signals) with and without the counterstained nucleus (blue). Green signals represent copies of chromosome 12 centromere (D12Z1). Normal cells have 2 copies of D12Z1 and sSMC cells have 3 copies of D12Z1.

We performed 3D Q-FISH to compare the telomere organization within both cell lines (normal and with sSMC) from the same patient (Figure 7). We also compared the results of the same cell line between the two patients. Normal nuclei cells with 2 copies of D12Z1 on (centromere of chromosome 12)(Karyotype 46,X_) were named 2G and the sSMC nuclei cells with 3 copies of D12Z1 (47,X_,+mar(12) as 3G.

Supplementary Table S1 shows the different cell lines and the Q-FISH results. The 2G cells from patient 1 showed higher *a/c* ratio (P=0.007) compared to 3G. We did not observe significant differences between Teloview parameters for 2G cells of patient 2 compared to 3G cells (Supplementary Table S1). The comparison of normal cell lines of both patients showed that 2G cells from patient 2 presented higher average intensity of signals (P<0.0001), total intensity of signals (P<0.0001), and higher *a/c* ratio (P<0.0001) compared with 2G cells of patient 1. Also, 3G cells from patient 2 presented higher average intensity of signals (P<0.0001), total intensity of signals (P<0.0001), and higher *a/c* ratio (P<0.0001) compared with 3G cells of patient 1.

## Discussion

Both patients evaluated in this study presented a mosaicism of cells with sSMC and normal cells. The sSMC was derived from chromosome 12 and involved the proximal region between the bands 12p11.21 and 12q13.11. In patient 1, the sSMC origin was characterized by FISH and confirmed by aCGH, but in patient 2, the aCGH was unable to detect the sSMC because of the low level of sSMC cells (below 18% in all analyzes performed). The technical limitations of aCGH in mosaic analysis, when the percentage of abnormal cells is lower than 25%, are well known. In those cases, aCGH is unable to detect abnormal cells reliably, while FISH can detect as low as 3% of abnormal cells by analyzing 100 cells (30). The structure of the two sSMC differed only by telomeric repeat region analyzed by FISH. Patient 2 sSMC was positive to clusters of telomeric repeats and negative in patient 1 sSMC. The telomere profile analysis showed that patient 1 telomeres were shorter than patient 2. The telomeric repeat clusters on the ends of sSMC from patient 1 may be too small to be visible in metaphase FISH analysis (31), but this does not mean that they were not present.

The parental origin of sSMC could be different in each case: the sSMC of patient 1 was inherited from her father (patient 2), whereas patient 2 sSMC were a *de novo*. The rate of *de novo* sSMC is approximately 70% compared with familial sSMC, approximately 30% (32). In this case, it is assumed that patient 1's zygote cell had the sSMC and that sSMC was lost through subsequent cell divisions generating the normal cell line and the mosaicism. There are two possible explanations for patient 2's *de novo* sSMC formation: 1) prezygotic or germinative origin with a post-zygote error; and 2) only post-zygotic origin (5).

The most accepted hypothesis for sSMC formation is a prezygotic or germinative origin, in which the primary driver for *de novo* SMCs is a non-disjunction at the maternal meiosis followed by a partial trisomy rescue in the trisomic zygote originating the supernumerary chromosome, through chromothripsis-like processes (5). The sSMC can be lost along subsequent divisions generating the normal cell line and the mosaicism. In the second possibility, after mis-segregation or nondisjunction between chromosomes in the normal zygote cell, a partial trisomy rescue occurs in an attempt to repair the numerical aberration and a part of the original trisomic chromosome remain in the form of sSMC that is kept over subsequent divisions, in mosaic formation with a normal cell line. Both hypotheses are possible, since sSMC cells were observed in all three analyzed tissues and these tissues originate from different germ layers. We presumed the presence of sSMC in germinal cells of patient 2.

In both patients, there were no significant differences in the frequency of sSMC cells in cultures of different durations, suggesting that the sSMC had no *in vitro* selection. Furthermore, for patient 1, the proliferation index was not significantly different between the two cell lines. This indicated that the cell line with sSMC and the normal cell line were proliferating at the same rate. However, the proliferation index of patient 2 indicated that his normal cells proliferated faster than cells with sSMC. Although no differences were observed in the proliferation index of the two cell lines of patient 1, the analysis of the telomere profile indicated that the normal cells were at a more advanced cell cycle phase. Also, both cell lines of patient 1 were at an earlier cell cycle phase than patient 2, suggesting that cells from patient 2 were proliferating faster than cells from patient 1.

According to Laurie et al. (19), cells with anomalous constitution can be selected *in vitro* and *in vivo* because they have different rates of proliferation and survival capacity. Although our data did not show *in vitro* selection of cells with sSMC, they showed that the presence of sSMC changes cell dynamics causing a lower proliferation rate. This suggests that sSMC cells take longer to complete the cell cycle in an attempt to correct the structural anomaly, or because the increase in the amount of genetic material caused by the presence of the sSMC, or because of the high energy and nutritional demand of cells with genomic imbalance. These factors probably have a higher impact *in vivo* contributing to the low frequency of cells with sSMC observed in the two patients.

Although they had the same sSMC, patient 1 had significantly more cells with sSMC than patient 2 at all culture times. Further, the clinical repercussions were significantly different, since only patient 1 had dysmorphia and developmental delay. According to Liehr et al. (2), the phenotypic repercussion is mainly influenced by the involved genomic segment, the parental origin, and the genetic background.

There was no significant difference between the sSMC frequencies in the different tissues of patient 1, meaning that the sSMC cells had a homogeneous distribution in the analyzed tissues, which probably reflected the distribution in other tissues of the body. In patient 2, a significant difference was observed in the distribution of sSMC cells between blood and the other tissues analyzed, indicating that the distribution of these cells was more heterogeneous and may be different in other body tissues such as the brain, which could explain the absence of phenotypic repercussions in this patient. In addition, patient 1 had significantly more cells with sSMC in all analyzed tissues, corroborating with results obtained by other analyses.

A higher frequency and homogeneous distribution of the cells with sSMC in patient 1 play a key role to explain the fact that this patient had phenotypic repercussions while patient 2 did not. The fact that patient 2 had more normal cells and less sSMC cells compared with patient 1 can also be associated with the age of the patients. In a 10-year follow-up study, Denes et al. (33) observed that the proportion of normal cells increases with time in women with Turner syndrome.

Epigenetic mechanisms such as imprinting and position effect can explain the difference in clinical repercussion, but none of the known imprinted or predicted genes on chromosome 12 are located between the bands present on the sSMC (12p11.21 and 12q13.11). According to Luedi et al. (34), failure to confirm imprinting does not eliminate the possibility that this gene may be imprinted.

Regarding SCE and chromosomal instability analysis, sSMC cells from patient 1 had significantly more SCE than normal cells. In contrast, patient 2 showed no significant differences between the two cell lines, suggesting that cells with sSMC from patient 1 showed higher instability compared to normal cells, but the amount of SCE in both cell lines in the two patients was not remarkable. In patient 2, it was not possible to tell which of the two cell lines was more unstable. In addition, it was not possible to predict which patient cells were more unstable by SCE analysis due to the sample size and the low frequency of the cells with sSMC.

The telomere analysis indicated a high telomere-related genomic instability in sSMC cells from patient 1 compared to the normal cells. Interestingly, both cell lines from patient 1 had shorter telomeres compared with cell lines from patient 2, which indicated high levels of genomic instability, since younger people are expected to have longer telomeres (35- [Bibr B36] 37). Short telomeres that fall below a critical length are hotspots for DNA damage resulting from illegitimate recombination and have been associated with chromosomal instability (38). Treff et al. (36) showed that aneuploidy can be associated with telomere shortening, since embryonic aneuploid cells have less telomeric DNA than embryonic euploid cells in the cleavage stage. In addition to the lower telomere-related genomic instability, the normal cells from patient 2 had the highest proliferation index, higher *a/c* ratio, and longer telomeres, which, together with the other results obtained here, indicated higher genetic stability of this patient's cells.

The highest instability is strongly associated with cell maintenance, loss, or replication dynamics. The high rate of post-zygotic chromosomal instability would intuitively imply that many other birth defects should occur. In *in vitro* fertilization (assisted reproduction), diploid blastomeres have a proliferative advantage over aneuploid cells (37,39). Thus, it is believed that aneuploid cells can be actively or passively eliminated or corrected (40). Passerini et al. (6) have also shown that the gain of a single chromosome increases genomic instability, as was seen in our results.

The differences found in sSMC structure reflect the complexity and the possibility of sSMCs evolution through generations, which may influence cell cycle dynamics. Taken together, our data could not explain if the presence of sSMC was responsible for the observed instability or if this instability already existed and contributed to the formation of the sSMC in patient 2. However, our data showed that sSMC cells have higher instability and a different cell cycle dynamic compared with normal cells. We also showed that these cells exhibit telomeric instability, which enables development of tumors and progression of tumor cells to adapt selectively to different environmental pressures resulting in intratumoral cell heterogeneity (9).

Our data provide a better understanding of sSMC stability and maintenance during development by indicating variables and different mechanisms associated with the phenotypic repercussions of rare structural imbalances. More detailed constitutional studies can contribute to the study of the sSMC evolution across generations.

## Supplementary Material

Click to view [pdf].
